# When Advocates are Forced to go Virtual: The Long-Term Care Ombudsman Response to COVID 19

**DOI:** 10.1093/geroni/igab046.3089

**Published:** 2021-12-17

**Authors:** H Wayne Nelson, F Ellen Netting, Mary W Carter, Bo Kyum Yang

**Affiliations:** 1 Towson University, Towson, Maryland, United States; 2 Virginia Commonwealth University, Richmond, Virginia, United States

## Abstract

This study explores strategies used by the nation’s Long-Term Care (LTC) Ombudsman Programs (LTCOP) to perform their grass roots, investigatory, sentinel defense advocacy during the near total COVID LTC lock out from March 13th 2020 through September 17th, when the “ban” was conditionally lifted. Our layered systematic searches by title, subject, and total text for unrestricted allusions to the LTCOP used the multi-disciplinary database Academic Search Ultimate that includes mass media. Selection criteria included print and broadcast news. Search keywords were “ombudsman” singly and with 10 other terms. This was augmented by reviewing the National LTC Ombudsman Resource Center (NORC) clearinghouse information website and by interviewing NORC staff. Resulting (172) media entries (92% print) were manually coded independently by a team of five, and iteratively reconciled according to a simple flat frame format to identify key words and associated themes. Four main LTCOP lock out strategies emerged: (1) virtual resident interventions (via phone, Skype, Zoom, in-facility allies); (2) public outreach (services provided, sharing COVID data and best practices, social isolation threats and mitigation efforts [window visits], need for volunteers); (3) systems advocacy (state/federal; CMS, legislative and other testimony about social isolation, CARES Act check problems, visitation issues; and (4) partnering with others (multi-agency planning groups, task forces, Zoom town halls, interstate information sharing). NORC interviews revealed that older LTCOP volunteers are seizing the COVID lock-out to retire undermining an already short-staffed network—so calls for volunteers were evident in about 25% of all stories regardless of any other focus.

